# Palliative medicine fellows attend to compassion fatigue using John Stone’s ‘Talking to the Family’

**DOI:** 10.1007/s40037-015-0169-9

**Published:** 2015-03-27

**Authors:** Hunter Groninger

**Affiliations:** Programme in Bioethics and Medical Humanities, University of Virginia (Charlottesville, Virginia), Capital Caring (Falls Church, Virginia), National Institutes of Health (Bethesda, Maryland), 3309 20th Road N, 22207 Arlington, Virginia USA

**Keywords:** Palliative medicine, Burnout, Compassion fatigue, Poetry, Self-care

## Abstract

For graduate medical education trainees, as well as contemporary practitioners, developing skills in recognizing compassion fatigue and practising self-care is vital to professional sustainability. The field of palliative medicine is no exception. In our fellowship programme, we use John Stone’s poem, ‘Talking to the Family,’ to engage trainees in a professional development workshop on personal experiences and strategies for self-care.

Contemporary medical practice, with its systemic challenges of complex reimbursement, increased documentation, shorter patient visit times and higher visit volumes, has become a ripe medium for career burnout and compassion fatigue [1]. For graduate medical education trainees, developing skills in recognizing compassion fatigue and practising self-care is vital to professional sustainability [2].

These issues are inherently relevant to the field of palliative medicine, where clinicians routinely participate in the emotionally intense care of the seriously ill [3]. To begin to address this, we require fellows in our palliative medicine training programme to participate in a faculty-led workshop on professional compassion fatigue and self-care. Our training programme supports 2–4 fellows who have trained in any primary medical speciality for a 1-year fellowship. The experience described here is drawn from two consecutive years of 4 fellows each year. Of these 8 fellows in total, 6 were internists and 2 were family physicians by training. Six fellows had joined our programme directly after residency training, and 2 had been in community-based practice, each for less than 5 years. Our faculty participants included 3 palliative medicine physician faculty, 2 of whom had been practising medicine for at least 10 years. This 3-h workshop takes place early in the academic calendar, usually around the end of the first quarter, and involves a collage of reflective writing exercises, dialogue about personal experiences, and didactic.

The pedagogical centrepiece of our workshop is John Stone’s poem, ‘Talking to the Family.’ [4] In this short work, a physician contemplates the dreadful family meeting he is about to undertake:

My white coat waits in the corner

like a father.

I will wear it to meet the sister

in her white shoes and organza dress

in the live of winter,

the milkless husband

holding the baby.

I will tell them.

They will put it together

and take it apart.

Their voices will buzz.

The cut ends of their nerves

will curl.

I will take off the coat,

drive home,

and replace the light bulb in the hall.

The poem is relatively simple in structure and theme but very rich in drama. Almost immediately, the fellows piece together the framework of a clinical story: a woman, present only by her relationships to others (‘the sister,’ ‘the husband,’ ‘the baby’) has died, perhaps in childbirth. This physician (referred to here as a male, although the poem itself does not delineate the physician’s gender) mentally rehearses a sequence of actions beginning in his office and ending at home.

After identifying subjects and themes in Stone’s poem, approximately 45 min of workshop time is dedicated to analyzing the key issues depicted. For our particular educational purposes, this is a poem about communicating bad news—a routine part of any palliative care practice. Our fellows seem to quickly empathize with the physician’s internal struggle to prepare for what will certainly be an emotionally difficult conversation. A discussion guided by physician faculty, focused solely on the written work itself, helps these fellows identify thematic, syntactic, and symbolic indicators of the emotional components to challenging family meetings. Through this dialogue, fellows seek to understand whether this physician/narrator is demonstrating compassion fatigue or appropriate empathy balanced with self-care strategies. Their conclusions (Table 1), recorded by this author during the workshop, underline the poem’s powerful obscurity—a reminder of the interplay between maintaining appropriate professional boundaries and emotional burnout.

**Table 1:** Observations by palliative medicine fellows regarding the ambiguity of emotional connection depicted in ‘Talking to the family’

Compassion fatigue	Empathy balanced with appropriate boundaries
He’s ‘talking *to*,’ not ‘talking *with*,’ the family	The white coat, symbolizing his professional role, protects him amidst emotional upheaval
He is not listening to their response (‘Their voices will buzz’)	White, the symbol of the family drama (shoes, dress, winter, milk), is reflected in his lab coat: he participates in the family drama
He’s only acting (putting on, taking off the white coat)	Wearing the white coat signifies the time to be actively present to the family
The light bulb at home is metaphorically burned out—like him	He actively replaces the light bulb
After an emotional encounter, he goes home to something mundane	After an emotional encounter, he is able to take care of himself professionally by moving on

After this guided discussion of the poem, fellows and faculty participate in a reflective writing exercise: each participant writes for 5 min about an emotionally provocative encounter with a patient, family member, or colleague. The goal of this exercise is simply to help participants begin to shift their attention from the poem to their own experiences. Examples from fellows have included a) repeated conversations with young parents about the imminent death of their newborn from a genetic abnormality, and b) communicating prognosis to a woman whose husband experienced a cardiac arrest and massive stroke.

Participants are encouraged, but not required, to share their writings, either by reading them directly or narrating these stories. For the next 45 min, through moderation by this author, fellows are asked to reflect on their emotions during these challenges and guided to identify, if appropriate, instances when compassion fatigue may have been at play. Individuals are also guided to reflect on steps they may have taken (or chose not to take) to mitigate compassion fatigue. From the examples noted above, the fellow who described repeat conversations about poor prognosis with young parents related experiencing a growing resistance on her part to visiting those parents, and tried to avoid the patient’s room; she also noted feelings of helplessness at work and wondered if she should stop practising medicine; she focused her self-care on spending more time with her own children and on journaling. The fellow who had a hard time communicating prognosis to a woman whose husband experienced cardiac arrest shared that she felt herself depersonalizing the patient and was disturbed by this, but found her situation improved after the patient was transferred to another facility; she expressed a wish to practise healthier self-care strategies.

Faculty participants are also encouraged to validate the challenges of dealing with emotionally provocative encounters by sharing their own professional experiences, and probing fellows as to current practices of self-care, or opportunities to do it more routinely. Using ‘Talking to the Family’ as a reference point, the conversation transitions to healthy strategies for self-care and maintaining emotional health in the workplace. Self-care techniques shared in the workshop have included physical (e.g. regular exercise, healthy diet, sleep hygiene), psychological (e.g. counselling, meditation), social (e.g. appropriate work/personal life boundaries, spending time with family/friends), and spiritual (e.g. religious traditions, ritual, nature walks) strategies.

In our workshop, Stone’s poem is used to promote reflection around emotionally charged clinical encounters and the impact they have on our personal and professional lives. The examples noted above reflect the acute care-focused content provided by our fellows; this is probably because these 8 fellows all came to fellowship from training or employment in acute care settings. However, it is likely that participants with more ambulatory or chronic care settings would have equally relevant experiences for discussion; one need only imagine or remember, for example, a difficult patient with chronic non-malignant pain or chronic non-adherence to disease management. The object of our exercise was simply to provoke dialogue about experiences of compassion fatigue, wherever and however they take place.

This workshop experience has drawn from physician trainees in a late stage of professional training, with enough clinical experiences and emotional insight to reflect internally and be comfortable sharing with peers and faculty. The small group setting promotes dialogue. This reflective exercise based on ‘Talking to the Family’ may certainly be possible in larger groups or with those in earlier stages of professional development; however, the same level of intimate reflection and dialogue may be less readily achievable. Whatever the audience or setting, our programme’s experience has found most crucial to explicitly set and maintain a tone of voluntary, non-judgmental active participation and dialogue. Participants with any level of clinical experience have encounters to share and to learn from others if the setting is respectful and safe. As can be so advantageous with the humanities, beginning the workshop with Stone’s poem helps set the stage for reflection by first focusing emotional themes on a work of literature before turning the gaze to oneself.

This article aims to describe a simple but effective pedagogical process for engaging learners in themes of compassion fatigue and self-care. Currently, our programme does not evaluate workshop participation; engagement in reflection and dialogue is the workshop’s own end goal. However, in our programme, the workshop and its content do herald ongoing conversations with individual fellows about their experiences of compassion fatigue and strategies for self-care for the remainder of their training year. While we do not employ measurable outcomes to this end, we aim to promote sustainability in clinical practice; open dialogue is a start to such sustainability.

Our palliative medicine fellows have universally expressed appreciation for Stone’s poem as a creative means to gain insight about emotional challenges in professional communication and the risks of developing compassion fatigue. They also welcome the poem as a springboard to more comfortable discussion with fellowship faculty about these themes; such an environment, ripe for dissecting emotionally difficult professional experiences, is uncommon in graduate medical training but seen by our fellows as highly valuable. Through the poet’s artistic lens, we acknowledge to our trainees the internal tensions that often arise when called to deliver bad news and the lifelong professional struggle we all experience to balance empathic communication and emotional self-care. Thus, the stage is better set for healthier professional development.
